# Genome Sequencing of 10 Helicobacter pylori Pediatric Strains from Patients with Nonulcer Dyspepsia and Peptic Ulcer Disease

**DOI:** 10.1128/genomeA.01488-14

**Published:** 2015-02-05

**Authors:** Alexandra Nunes, Raquel Rocha, Filipa F. Vale, Luís Vieira, Daniel A. Sampaio, Ricardo Dias, João P. Gomes, Mónica Oleastro

**Affiliations:** a Bioinformatics Unit, Department of Infectious Diseases, National Institute of Health, Lisbon, Portugal; b National Reference Laboratory of Gastrointestinal Infections, Department of Infectious Diseases, National Institute of Health, Lisbon, Portugal; c Host-Pathogen Interactions Unit, Research Institute for Medicines (iMed-ULisboa), Institute of Molecular Medicine, Faculty of Pharmacy, University of Lisbon, Lisbon, Portugal; d Innovation and Technology Unit, Department of Human Genetics, National Institute of Health, Lisbon, Portugal; e Center for Biodiversity, Functional and Integrative Genomics, Faculty of Sciences, University of Lisbon, Lisbon, Portugal

## Abstract

We present draft genome sequences of 10 Helicobacter pylori clinical strains isolated from children. This will be important for future studies of comparative genomics in order to better understand the virulence determinants underlying peptic ulcer disease.

## GENOME ANNOUNCEMENT

Long-term infection with Helicobacter pylori is the main cause of peptic ulcer disease (PUD), the most common severe condition associated with this bacterium. It affects 15–20% of infected adults, causing them considerable morbidity and mortality (1). Considering the high prevalence rates of infection worldwide, PUD is a major public health problem with high annual costs of treatment. In children with no other etiology for the disease, this rare event occurs shortly after infection and is thus a good model for the search of virulence biomarkers.

In this study, 10 strains were isolated from antral biopsies collected from Portuguese children with ages ranging from 7 to 15 years. Five specimens were obtained from nonulcer dyspepsia cases, while the remaining five were isolated from children exhibiting gastric or duodenal ulceration (Table 1). Briefly, biopsies were ground with a tissue homogenizer and inoculated onto both non- and selective media at 37°C under a microaerobic environment, for up to 14 days of incubation. H. pylori identification was performed according to conventional tests: colony and Gram stain morphologies, catalase, oxidase, and hydrolysis of urea. For each strain, total DNA was extracted using the QIAmp DNA minikit according to the manufacturer’s instructions. After fragmentation, dual-indexed Illumina libraries of genomic DNA were prepared with the Nextera XT Index kit and then subjected to cluster generation and paired-end sequencing (2 × 250 bp or 2 × 300 bp) on a MiSeq Illumina platform. The resulting mean depth of coverage ranged from 39- to 156-fold (Table 1). After trimming with the FASTX tool (http://hannonlab.cshl.edu/fastx_toolkit), high-quality reads were *de novo* assembled using Velvet version 1/2/10 (2). Contigs were visually inspected with the Tablet version 1.14.04.10 graphical viewer (3) and properly corrected. Annotation was performed using the RAST server (4) and the NCBI Prokaryotic Genomes Annotation Pipeline version 2.3. Putative plasmids were identified by checking contigs >1,000 bp with high depth of coverage and evidence of circularity. Search for putative prophages was performed using the PHAge Search Tool (PHAST) (5).

**TABLE 1:** Description of the 10 H. pylori strains and of the Portuguese children from whom each was isolated^*a*^

Strain	Strain features						Patients^*b*^		
	Yr of isolation	Accession no. (no. of contigs)	Total size (bp)	Mean depth coverage (per base)	Putative plasmid (size)	No. of CDSs (pseudogenes)	Age (yr)	Gender	Clinical symptom
499/02	2002	JTDG00000000 (73)	1,666,743	126×	No	1,518 (66)	11	M	GU
1089/03	2003	JSUY00000000 (62)	1,587,159	134×	No	1,448 (75)	10	M	DU
1152/04	2004	JSUZ00000000 (59)	1,588,646	156×	No	1,449 (77)	10	M	DU
1198/04	2004	JSXT00000000 (57)	1,618,345	72×	pHPY1198 (2,794 bp)	1,485 (64)	15	M	DU
1846/05	2005	JSXV00000000 (58)	1,635,807	52×	pHPY1846 (3,161 bp)	1,500 (70)	13	M	DU
173/00	2000	JSXX00000000 (79)	1,562,065	32×	pHPY173 (4,194 bp)	1,438 (71)	14	M	NUD
207/99	1999	JSXU00000000 (47)	1,544,449	142×	No	1,395 (73)	7	F	NUD
228/99	1999	JSXY00000000 (54)	1,614,472	39×	pHPY228 (4,194 bp)	1,487 (59)	8	M	NUD
655/99	1999	JSXB00000000 (45)	1,615,666	130×	No	1,475 (63)	11	M	NUD
1786/05	2005	JSXW00000000 (41)	1,616,052	50×	pHPY1786 (9,960 bp)	1,467 (77)	11	M	NUD

a All strains are indicated by their collection number. All patients were from Portugal.

b M, male; F, female; NUD, nonulcer dyspepsia; DU, duodenal ulcer; GU, gastric ulcer.

Overall, the draft genomes of the 10 H. pylori strains varied from 1.54 to 1.67 Mb, with an average G+C content of 39% (Table 1). All ulcerogenic strains possessed a complete *cag* pathogenicity island, revealing a highly conserved gene content and gene order. Five different putative plasmids (sizes 2,794–9,960 bp), which are apparently cryptic based on BLAST results, were found for two duodenal ulcer and three nonulcer strains. With the exception of pHPY1198, all plasmids likely display more than 10 copies *per* chromosome. Two of the ulcerogenic strains, 1846/05 and 499/02, were found to carry a putative intact prophage of 16.7 Kb (GC% 36.7) and 15.3 Kb (GC% 37.4), respectively, both comprising 23 CDSs.

In order to better understand the genetic diversity among H. pylori strains that cause peptic ulcer disease in children, an in-depth comparative analysis between these ulcerogenic and nonulcerogenic draft genomes is under way and will be published in a subsequent report.

### Nucleotide sequence accession numbers.

The draft genome sequences of each H. pylori strain were deposited in GenBank under the accession numbers listed in Table 1.
